# Shifting Dominant Air Pollution Sources in Sub-Saharan Africa’s Changing Urban Environment: Analysis of Elemental Composition and Source Contributions of Ambient Fine Particulate Matter (PM_2.5_) Pollution in Accra, Ghana

**DOI:** 10.1021/EHP.6c00323

**Published:** 2026-10-09

**Authors:** Carissa L. Lange, Aaron J. Specht, Abosede S. Alli, Sierra N. Clark, James Nimo, Thomas Grier, Raji Balasubramanian, Michael Brauer, Allison F. Hughes, Majid Ezzati, Raphael E. Arku

**Affiliations:** † Department of Environmental Health Sciences, 541939 University of Massachusetts, Amherst, Massachusetts 01003, United States; ‡ School of Health Sciences, 311308 Purdue University, Lafayette, Indiana 47907, United States; § Department of Population Health & Policy, School of Health and Medical Sciences, City St George’s, University of London, London EC1V 0HB, UK; ∥ Department of Physics, University of Ghana, Legon, Accra 00233, Ghana; ⊥ Department of Environmental & Sustainable Engineering, State University of New York, Albany, New York 12222, United States; # Department of Biostatistics & Epidemiology, University of Massachusetts Amherst, Amherst, Massachusetts 01003, United States; 7 School of Population and Public Health, The University of British Columbia, Vancouver, British Columbia V6T 1Z3, Canada; 8 Institute for Health Metrics and Evaluation, University of Washington, Seattle, Washington 98195, United States; 9 Department of Epidemiology and Biostatistics, School of Public Health, Imperial College London, London W2 1PG, UK; 10 Regional Institute for Population Studies, University of Ghana, Accra 00233, Ghana; 11 MRC Centre for Environment and Health, School of Public Health, Imperial College London, London W2 1PG, UK; 12 Imperial Global Ghana, Accra LG25, Ghana

## Abstract

Urban and economic expansion across Sub-Saharan Africa (SSA) may be shifting the relative contributions of dominant ambient air pollution sources, influencing both pollutant concentrations and composition, along with their associated health risks. However, city-scale data on the composition and source contributions are scarce, hindering source- and sector-specific policy interventions. Using samples from a large-scale, citywide monitoring campaign, we characterized the elemental composition and source contributions of PM_2.5_ pollution across the Accra metropolis, a major SSA city. From April 2019 to June 2020, we collected over 600 weekly gravimetric (filter-based) PM_2.5_ samples from 146 unique sites spanning diverse land-use across the city. Filters were analyzed for several elements using energy dispersive x-ray fluorescence (ED-XRF), and positive matrix factorization (PMF) was applied to identify major sources, and changes in their relative contributions, at four legacy sites from a decade prior (2007-2008). Concentrations of elements associated with biomass burning (K, Br, Pb) and vehicle emissions (Ti, Fe, Cu, Zn) were highest in high-density residential and heavily trafficked areas, respectively. There was strong temporal variation in several elements (Al, Si, Fe, Ti, K, Ca, Mn) across seasons, with elevated concentrations during the dusty Harmattan season, when crustal dust accounted for 52-68% of total elemental PM_2.5_ mass. In the non-Harmattan period, we identified 4-6 major sources including biomass burning (comprising 11-32% of total PM_2.5_ elemental mass), traffic emissions and road dust (9-36%), solid and electronic waste combustion (0-35%), construction dust (0-30%), crustal dust (11-47%), and sea salt (8-67%). Compared to a decade prior, the contribution from biomass burning to overall PM_2.5_ has decreased by ∼16% (95% CI: 5.2-25.8; *p-value* = 0.017). Contrarily, the composite traffic/road dust factor remained stable, though increasing traffic emissions may have been offset by declining road-dust contributions. Most notably, contributions from solid and electronic waste burning have risen significantly (mean difference = 23%; 95% CI: 15.9-30.0; *p-value* = 0.002) to levels approaching those of biomass burning and traffic-related sources at legacy sites. Our findings indicate that household biomass use remains a dominant source of PM_2.5_ in Accra, although its relative contribution appears to have declined. This reduction may be partially offset by increasing contributions from solid and electronic waste burning in many locations. These shifts in PM_2.5_ composition and relative source contributions underscore the need for source- or sector-specific policy actions in the region.

## Graphical abstract

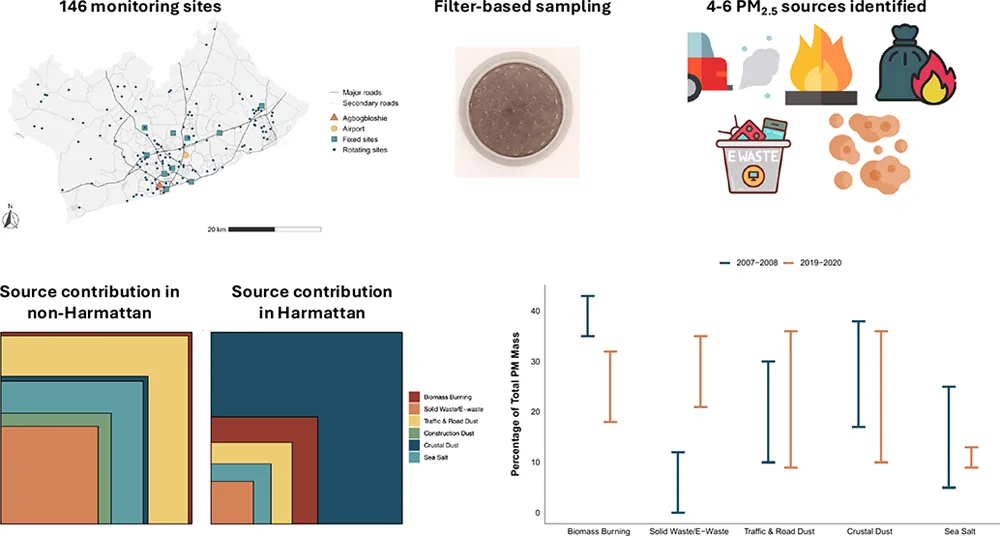

## 1 Introduction

Urban and economic expansion across Sub-Saharan African (SSA) is driving shifts in energy use, vehicle fleets, waste management, and regulatory policies that are collectively transforming the region’s environmental landscape.^1,2^ These transitions have important implications for ambient fine particulate matter (PM_2.5_), a leading global environmental risk factor for cardiovascular, respiratory, and other chronic diseases, by altering both the concentrations and composition of PM_2.5_ to which urban residents are exposed.^3^ While traditional sources such as biomass combustion and road traffic remain important contributors to the air pollution mixture in the region, SSA cities also experience unique sources, including informal and small-scale industrial activities in residential areas, trash and electronic waste burning, resuspended dust from unpaved roads, and seasonal regional dust storms.^4^ This diversity of sources creates a more complex source-emissions environment in rapidly growing SSA cities than has typically been documented in high-income settings, highlighting the need for source-resolved evidence, rather than PM_2.5_ mass concentration alone, to identify which emission sources are driving health-relevant exposures.

PM_2.5_ sources and their relative contributions may also be shifting amid this ongoing transition. Households are transitioning from reliance on solid fuels to relatively cleaner options such as Liquified Petroleum Gas (LPG).^3^ At the same time, the number of motor vehicles in circulation continues to rise and is projected to double due to growing urban demand, with a fleet that is becoming newer on average but still contributing substantially to ambient traffic-related emissions.^5^ A further and increasingly urgent concern in SSA cities is the generation of toxic air pollutants from the unregulated burning of imported electronic waste (e-waste). Of the 53.6 million metric tons of e-waste generated worldwide in 2019, nearly 40% was processed at the Agbogbloshie site in Accra, Ghana,^6^ contributing to elevated air pollution levels and complex pollutant mixtures^7,8^ that have been linked to well-documented health risks, including elevated exposure to lead and other toxic metals among nearby residents.^9^ Taken together, these dynamics suggest that while the dominance of traditional biomass sources may be receding, emerging urban sources, particularly traffic, solid waste, and e-waste burning, may play an increasingly important role in shaping PM_2.5_ composition and its associated health risks in the region, with important implications for future air quality management and exposure reduction strategies.

Despite the complex and rapidly evolving emissions landscape in SSA, source apportionment studies remain scarce, and evidence on how PM_2.5_ sources are changing as cities urbanize is essentially absent from the peer-reviewed literature. In this study, we characterized elemental composition of PM_2.5_ pollution in Accra, Ghana, one of SSA’s fastest growing metropolises, using samples from a large-scale, citywide measurement campaign. We applied positive matrix factorization (PMF) to the elemental data to identify the major sources contributing to PM_2.5_ pollution in the city. Additionally, we examined changes in elemental composition and source contributions over roughly a decade by comparing our findings with a smaller earlier study conducted in the city in 2007–2008. To our knowledge, this represents one of the largest and most spatially resolved assessments of PM_2.5_ elemental composition and source apportionment conducted in an SSA city to date and is among the first to empirically examine decade-scale changes in source contributions in the region. While this study focuses on Accra, the underlying dynamics (i.e. rapid urbanization, growing vehicle fleets, and the rise of informal waste and e-waste burning as urban PM_2.5_ sources) are shared by many rapidly growing low- and middle-income cities. Thus, we anticipate that these findings will inform air quality management and exposure-reduction policies in Accra while also offering a transferable framework and evidence base for source-resolved PM_2.5_ characterization in similarly under-monitored urban settings.

## 2 Methods

### 2.1 Study Location

Our study is focused on the Greater Accra Metropolitan Area (GAMA), the industrial and administrative center of Ghana and one of the fastest growing metropolises in SSA. The GAMA covers roughly 1500 km^2^ and contains nearly six million residents. It comprises 25 administrative districts, including the Accra Metropolitan Area (AMA) at its core, and the port city Tema Metropolitan Area (TMA) to the east.^10^ The districts adjoining AMA and TMA consist of densely populated residential neighborhoods with heavy traffic, whereas those on the outskirts are predominantly peri-urban areas and emerging communities with significant construction activities. As Ghana’s capital and largest city, Accra has become a hub for business, technology, communications and education. However, large socioeconomic inequalities persist, particularly in housing and potential exposure to environmental health risks. Residents in densely populated communities are generally lower income earners, with most in shared compound houses relying on biomass for household cooking and for street food preparation. Trash burning and informal industrial activities are more common in densely populated communities. In contrast, higher income earners live in sparsely populated neighborhoods, consisting of modern low-rise homes on large plots of land where gas and electricity are the primary cooking fuels.^11^

The city experiences two major seasons: a rainy season (May-September) and a dry, dusty Harmattan season influenced by north-easterly trade winds from the Sahara Desert. Consequently, particle pollution levels are substantially elevated during the Harmattan period. Average temperatures and relative humidity range between 24°C-33°C (75-92°F) and 77%–85%, respectively.^10^

### 2.2 Study Design

From April 2019 to June 2020, weekly integrated gravimetric (filter-based) PM_2.5_ samples were collected at 146 sites across the geographic extent of the GAMA. The monitoring sites comprised of 10 ‘fixed’ sites, which were sampled weekly for the entire year, and 136 ‘rotating’ sites, which were sampled for 7 days. This resulted in 654 samples collected over the measurement period. An additional 27 duplicate samples and 28 field blanks were also collected at the rotating sites. The blanks were analyzed for elemental concentrations, and the average concentration of each element was used to correct the corresponding field samples.

Figure 1 shows a map of GAMA with the locations of the fixed and rotating sites. The fixed sites were purposefully selected based on population density, road traffic and networks, and neighborhood socioeconomic status. Traffic-dominant sites included N1 West (N1W), Tema Motorway (TMW) and Asylum Down (AD); Jamestown (JT) and Nima (NM) are low-income, densely populated neighborhoods with high biomass use; Taifa (TF) and Ashaiman (ASH) are emerging neighborhoods; Labadi (LA) is an indigenous community located along the ocean coast; East Legon (EL) is a high-income residential neighborhood that now also hosts large corporate, commercial and small business ventures, and is adjacent to the University of Ghana campus site (UGH), which served as a peri-urban or background site. Four of these sites (AD, JT, NM and EL) were also sampled in an earlier (2007-2008) air pollution study.^12^ The rotating sites were selected using a random sampling approach across land use classes and inside/outside the AMA.

**Figure 1:**
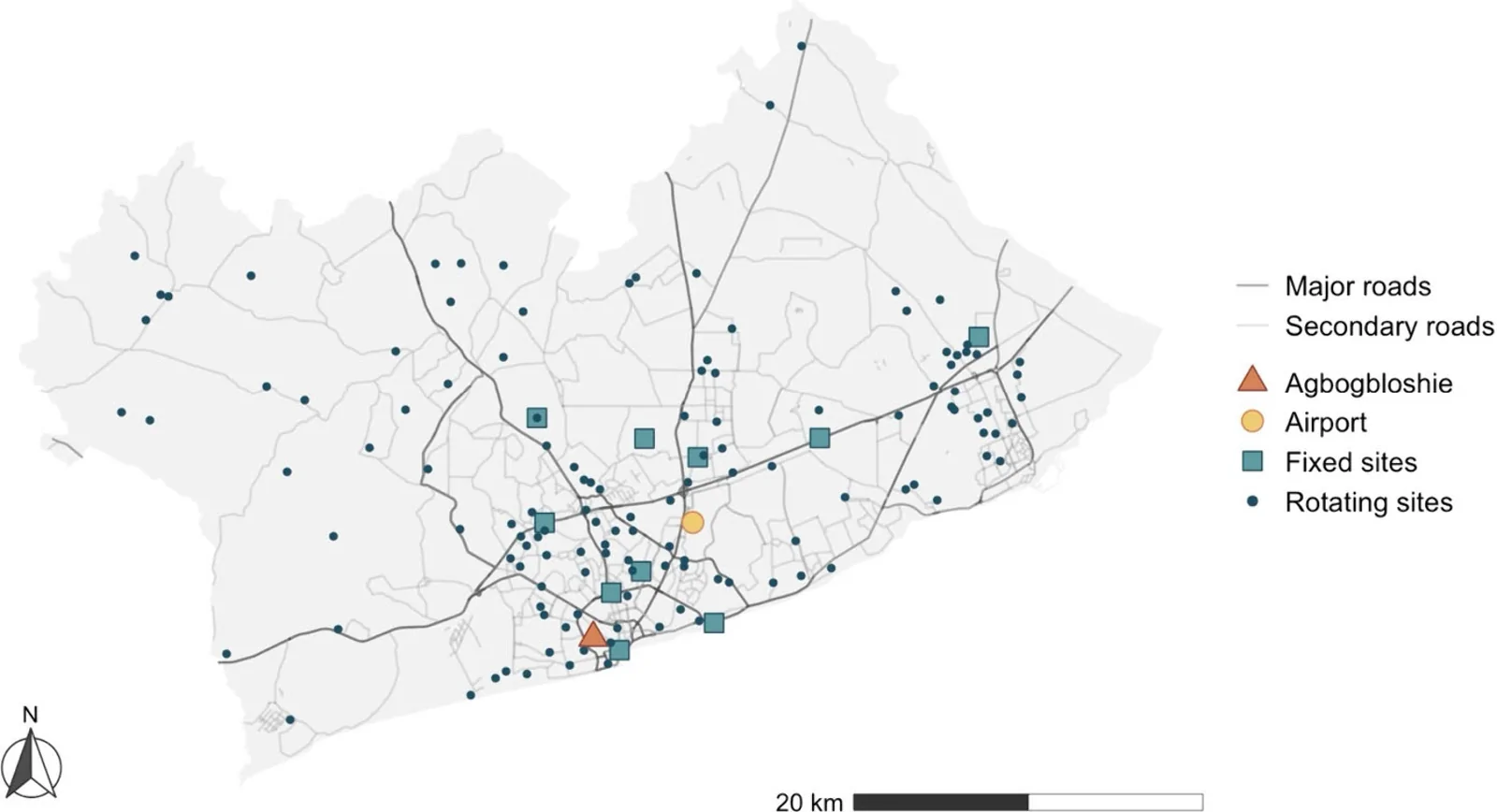
Map of the GAMA depicting year-long (fixed) and week-long (rotating) monitoring locations. Major and secondary roads are from OpenStreetMap (downloaded 2019) and the GAMA boundary from Ghana Statistical Service.

Each of the 146 monitoring sites were classified as one of four land-use categories: commercial, business, and industrial (CBI), representing neighborhoods with large buildings used for commercial, industrial, office, or warehouse purposes; high-density residential, representing neighborhoods with small, crowded, irregular buildings and narrow, unpaved roads typical of shanty towns and slums; medium/low-density residential, representing neighborhoods with small, planned buildings characteristic of formal residential areas; and peri-urban background, representing areas dominated by vegetation (e.g., dense forest), barren land (e.g., sand or soil), or bodies of water.^13^ Additional details of the overall study design have been described elsewhere.^13^

### 2.3 Field Data Collection

Weekly integrated gravimetric (filter-based) PM_2.5_ concentrations were collected using Ultrasonic Personal Aerosol Samplers (UPAS) fitted with 37 mm Teflon filter media. The UPAS sampled at 1 liter per minute at a 50% duty cycle. The monitors were placed in protective cases and fastened to metal poles approximately 4 meters above the ground. Filters were excluded from our analysis if they failed to meet our pre-specified inclusion requirements that the UPAS had to operate for ≥75% of the measurement period (i.e., 5 of 7 days) and with an average flow rate within 10% of the intended rate (1 L/min). All filters were weighed pre- and post-sampling using a MTL AH500 automated robotic scale at the University of British Columbia, and the net weight was used to compute the mass concentration. Additional information on the filter handling measurement methods has been previously published elsewhere.^14^

### 2.4 Elemental Analysis

The elemental concentrations of 603 filters were quantified using the Epsilon 4 ED-XRF instrument (Malvern PANalytical) at Purdue University. A total of 45 elements were analyzed: sodium (Na), magnesium (Mg), aluminum (Al), silicon (Si), phosphorous (P), sulfur (S), chloride (Cl), potassium (K), calcium (Ca), scandium (Sc), titanium (Ti), vanadium­(V), chromium (Cr), manganese (Mn), iron (Fe), cobalt (Co), nickel (Ni), copper (Cu), zinc (Zn), gallium (Ga), germanium (Ge), arsenic (As), selenium (Se), bromine (Br), rubidium (Rb), strontium (Sr), yttrium (Y), zirconium (Zr), niobium (Nb), molybdenum (Mo), rhodium (Rh), cadmium (Cd), tin (Sn), antimony (Sb), palladium (Pd), tellurium (Te), iodine­(I), cesium (Cs), barium (Ba), lanthanum (La), cerium (Ce), tungsten (W), gold (Au), mercury (Hg), and lead (Pb). However, P, Ga, As, Zr, Rh, Cd, Sn, Sb, Pd, Te, I, Cs, W, Au, Hg, V, Ba, Ce, Cs, and Se were excluded from the final analysis, as more than 75% of their measured concentrations were below the method detection limit (MDL). Each sample was measured for 24 min under varying x-ray conditions optimized for each of the elements included. Deposition area and sampled volume were used to convert mass loading (μg/cm^2^) to mass concentrations (ng/m^3^). To account for potential field contamination, samples were corrected using information from the field blanks.

### 2.5 Source Identification and Contributions

Source apportionment was conducted using the US EPA’s Positive Matrix Factorization (PMF) model (version 5.0). PMF is a statistical method that computes the best-fitting source profiles and their contributions by minimizing the sum of the squared residuals, weighted by the uncertainty of element concentrations.^15^ We calculated uncertainty values for each sample based on the MDL (Table S1) and an assumed error rate of 10% for elements.^16,17^ For concentrations at or below the limit of detection, values were replaced with the (MDL)/2 and uncertainty was calculated as *Unc* = 5/6*MDL*. For concentrations above the MDL, uncertainty was calculated as

$$Unc=\sqrt{{\left(0.1\times C\right)}^{2}+{\left(0.5\times MDL\right)}^{2}}$$

While PMF accounts for variations in pollution sources over time and across locations, it assumes that source profiles remain relatively consistent within a given model. Thus, to account for seasonal differences, PMF analyses were conducted separately for the Harmattan and non-Harmattan periods at each of the 10 fixed sites, as the dusty Harmattan period was expected to produce substantial shifts in the source profiles relative to the rest of the year. The shorter duration of the Harmattan season resulted in fewer samples per site (n = 12-16 samples per fixed-site Harmattan model, compared with n = 33-35 samples per fixed-site non-Harmattan model); we nonetheless retained seasonal stratification to avoid obscuring important differences in sources composition between periods, while flagging models with lower sample sizes for cautious interpretation below. In addition to the fixed monitoring sites, source apportionment was performed for the four broad site categories using data from the rotating sites (CBI, high-density residential, medium/low-density residential, and peri-urban background), in efforts to develop a more generalizable understanding of PM_2.5_ sources across the city (n = 11-24 samples per site category model). Because sites classified as medium/low-density residential varied substantially in local source features, this category was further split into (1) sites with roadside cooking, trash burning, or proximity to a medium-sized road, and (2) sites without these features, and located along minor roads only. PMF analysis of rotating-site filters was conducted only for the non-Harmattan period, as too few samples were collected at these sites during Harmattan to support separate models. Finally, we conducted two additional PMF models combining all fixed- and rotating-site data into a single pooled model for each season (n = 431 non-Harmattan; n = 172 Harmattan). In summary, we modeled: (i) each of the 10 fixed sites separately by season, (ii) the rotating sites by site type for the non-Harmattan period only, and (iii) the full, pooled dataset separately by season.

Each PMF model was initialized with 20 base runs, followed by 100 bootstrap (BS) runs to assess the stability of resolved factor profiles and the uncertainty of source contributions. The optimal number of factors was determined using a combination of BS diagnostics, goodness-of-fit statistics (Q values), and prior knowledge of regional PM_2.5_ sources. PMF models with 4-7 factors were evaluated, consistent with a previous study in the region that identified approximately 4-6 major source types.^12^ Model selection was guided by Q statistics (Q_robust_, Q_true_, and Q_true_/Q_exp_), with values closer to 1 indicating better fit and values substantially greater than or less than 1 indicating potential underfitting or overfitting, respectively. For most non-Harmattan models, the solution with the lowest Q was retained. However, some 7-factor solutions showed evidence of source splitting (e.g., a single crustal or biomass-burning factor separating into multiple factors), suggesting potential overfitting. In these cases, a more parsimonious solution was selected despite marginally higher Q values. To facilitate season comparisons, Harmattan models were, where feasible, constrained to match the factor structure identified for the non-Harmattan season at the same site or site group. This occasionally required accepting a solution with slightly higher Q values in order to preserve consistent, interpretable source categories across seasons. Sample sizes, Q statistics, and bootstrap factor-mapping results for all final selected models are presented in Table S2.

Signal-to-noise (S/N) ratios for all species included in the PMF models are reported in Table S3. For fixed site models, S/N ratios were averaged across the 10 monitoring sites to generate season-specific summaries for Harmattan and non-Harmattan periods. For rotating-site models, which utilized only non-Harmattan data, S/N ratios were averaged across the five site groupings. For the pooled, full-dataset models, S/N ratios were reported directly for each model. Following U.S. EPA guidance, species were considered “Bad” when S/N ratio was <0.5 and “Weak” when the S/N ratio was ≥ 0.5 but <1.^18^

Source contributions were estimated from the apportioned elemental mass. For each factor, the concentrations of all elemental species assigned to that factor were summed to obtain the total elemental mass attributable to the factor. The relative contribution of each factor was then expressed as a proportion of the total summed elemental mass across all factors. Reported source contributions therefore represent the percentage of measured elemental PM_2.5_ mass explained by each PMF factor, not the percentage of total gravimetric PM_2.5_ mass.

### 2.6 Methodological Comparison with a Prior Accra Source Apportionment Study

In a 2007-2008 study in Accra within the AMA, Zhou and colleagues conducted source apportionment of PM_2.5_ samples from four neighborhoods spanning a range of socioeconomic status and source features: Asylum Down (AD), East Legon (EL), Jamestown (JT), and Nima (NM).^12^ Our current study included these same four sites, enabling us to examine changes overtime at a consistent set of locations. However, there are several notable methodological differences between the two studies that warrant consideration. Both studies characterized elemental composition using ED-XRF analysis of particulate matter collected on Teflon filters and applied PMF to the resulting elemental mass concentrations to identify and quantify PM_2.5_ sources. However, our study used the Epsilon 4 ED-XRF instrument, whereas Zhou et al. used a Shimadzu EDX-700HS spectrometer. In addition, our study measured an expanded suite of elements, retaining 26 elements for source apportionment compared with 18 elements in the prior study, reflecting advances in ED-XRF capabilities. Accordingly, changes in source contributions observed between studies should be interpreted cautiously in the context of these methodological differences.

## 3 Results

### 3.1 Elemental Composition of PM_2.5_ Pollution

Across all site types, ten elements (S, Mg, Cl, Si, K, Fe, Ca, Na, Al, and Ti) constituted the largest proportion of the total elemental PM_2.5_ mass (Table 1). Concentrations of biomass and waste burning tracers (K, Br, Pb) were elevated primarily at high-density residential sites, while elements indicative of traffic emissions and road dust (Fe, Cu, Zn, Ti) were more abundant at sites near major roadways. The proportion of Na and Cl in elemental PM_2.5_ mass was highest at coastal sites influenced by frequent sea spray, while elements characteristic of crustal material (Al, Si, Mg, Ti) were most prominent at peri-urban background sites. Additionally, we observed pronounced temporal variation in several elements (Al, Si, Fe, Ti, K, Ca, Sc, Mn, Co, Rb, Sr), with their concentrations elevated 2-4 times during the dusty Harmattan season (Figure 2).

**Table 1:** Mean Concentrations of Total PM_2.5_ Mass and Elemental Constituents Across All Sites by Site Type during the Non-Harmattan Months^a^

**Element**	**Commercial** (n = 18)	**Traffic** (n = 103)	**High-density** (n = 92)	**Low-density** (n = 135)	**Ocean** (n = 35)	**Peri urban Background** (n = 48)
Mass^a^	21 ± 4	25 ± 5	24 ± 7	19 ± 6	21 ± 9	13 ± 15
Al	137 ± 164 (0.66%)^b^	143 ± 233 (0.56%)	108 ± 218 (0.45%)	172 ± 263 (0.92%)	73 ± 134 (0.32%)	163 ± 243 (0.95%)
Si	542 ± 348 (2.6%)	759 ± 505 (3.0%)	588 ± 597 (2.4%)	712 ± 621 (3.8%)	524 ± 408 (2.5%)	707 ± 644 (4.9%)
S	1168 ± 420 (5.6%)	1425 ± 586 (5.6%)	1421 ± 631 (5.9%)	1333 ± 514 (7.1%)	1559 ± 821 (7.7%)	1160 ± 460 (9.4%)
Na	204 ± 294 (1.0%)	227 ± 142 (0.89%)	219 ± 122 (0.91%)	205 ± 102 (1.1%)	1004 ± 728 (4.8%)	186 ± 92 (1.6%)
Cl	669 ± 754 (3.2%)	325 ± 669 (1.3%)	492 ± 505 (2.0%)	274 ± 441 (1.5%)	2755 ± 1621 (13.2%)	170 ± 236 (1.5%)
Mg	563 ± 647 (2.7%)	1114 ± 1657 (4.4%)	1117± 1669 (4.7%)	1105 ± 1490 (5.4%)	1133 ± 1848 (5.7%)	867 ± 1393 (7.4%)
K	522 ± 204 (2.5%)	620 ± 247 (2.4%)	1090 ± 450 (4.5%)	594 ± 209 (3.2%)	576 ± 319 (2.9%)	497 ± 182 (3.9%)
Fe	401 ± 281 (1.9%)	535 ± 264 (2.1%)	347 ± 518 (1.4%)	411 ± 355 (2.2%)	214 ± 242 (1.02%)	301 ± 292 (2.09%)
Ca	358 ± 224 (1.7%)	427 ± 179 (1.7%)	420 ± 528 (1.7%)	343 ± 246 (1.8%)	503± 534 (2.2%)	214 ± 171 (1.6%)
Ti	134 ± 84 (0.65%)	184 ± 122 (0.72%)	154 ± 192 (0.64%)	182 ± 166 (0.97%)	105 ± 94 (0.52%)	154 ± 140 (1.06%)
Zn	61 ± 69 (0.29%)	33 ± 22 (0.13%)	33 ± 22 (0.14%)	27 ± 31 (0.15%)	24 ± 26 (0.12%)	15 ± 12 (0.12%)
Br	20 ± 26 (0.10%)	20 ± 17 (0.08%)	26 ± 16 (0.11%)	11 ± 5 (0.06%)	19 ± 9 (0.10%)	7 ± 5 (0.05%)
Sc	18 ± 15 (0.09%)	21 ± 11 (0.08%)	22 ± 40 (0.09%)	17 ± 17 (0.09%)	28 ± 38 (0.12%)	9 ± 9 (0.06%)
Cu	11 ± 6 (0.05%)	28 ± 26 (0.11%)	20 ± 20 (0.08%)	16 ± 17 (0.09%)	20 ± 22 (0.10%)	13 ± 11 (0.12%)
Pb	14 ± 16 (0.07%)	9 ± 7 (0.03%)	12 ± 6 (0.05%)	16 ± 15 (0.04%)	6± 4 (0.03%)	4 ± 2 (0.03%)
Cr	11 ± 6 (0.05%)	17 ± 21 (0.07%)	14 ± 10 (0.06%)	4 ± 15 (0.08%)	16 ± 13 (0.10%)	11 ± 10 (0.08%)
Co	10 ± 7 (0.05%)	13 ± 7 (0.05%)	9 ± 13 (0.04%)	10 ± 9 (0.05%)	6 ± 6 (0.03%)	8 ± 7 (0.05%)
Nb	4 ± 1 (0.02%)	4 ± 3 (0.02%)	4 ± 3 (0.02%)	3 ± 3 (0.02%)	4 ± 3 (0.02%)	4 ± 3 (0.03%)
Mo	3 ± 2 (0.02%)	4 ± 3 (0.02%)	4 ± 3 (0.02%)	4 ± 3 (0.02%)	4 ± 2 (0.02%)	4 ± 3 (0.04%)
Sr	2 ± 1 (0.01%)	2 ± 1 (0.01%)	2 ± 3 (0.01%)	2 ± 1 (0.01%)	3 ± 2 (0.02%)	2 ± 1 (0.02%)
La	2 ± 2 (0.01%)	3 ± 4 (0.01%)	3 ± 4 (0.01%)	3 ± 5 (0.02%)	3 ± 4 (0.02%)	4 ± 4 (0.03%)
Mn	2 ± 1 (0.01%)	3 ± 1 (0.01%)	1 ± 2 (0.01%)	2 ± 2 (0.01%)	2 ± 2 (0.01%)	2 ± 2 (0.01%)
Y	3 ± 1 (0.01%)	3 ± 2 (0.01%)	3 ± 2 (0.01%)	3 ± 2 (0.01%)	3 ± 2 (0.02%)	3 ± 2 (0.03%)
Rb	1 ± 1 (<0.01%)	1 ± 1 (<0.10%)	1 ± 1 (0.01%)	1 ± 1 (<0.01%)	1 ± 1 (<0.01%)	1 ± 1 (0.01%)
Ge	1 ± <1 (<0.01%)	1 ± 1 (<0.01%)	1 ± <1 (<0.01%)	1 ± <1 (<0.01%)	1 ± 1 (<0.01%)	1 ± <1 (0.01%)
Ni	<1 ± <1 (<0.01%)	1 ± 1 (<0.01%)	<1 ± <1 (<0.01%)	<1 ± <1 (<0.01%)	<1 ± <1 (<0.01%)	1 ± <1 (<0.01%)

a Concentrations are in ng/m^3^ except for total mass, which is in μg/m^3^.

b Mean ± standard deviation. Numbers in parentheses show the proportion of total mass.

c Sample size (n) describes the total number of filters per category.

**Figure 2:**
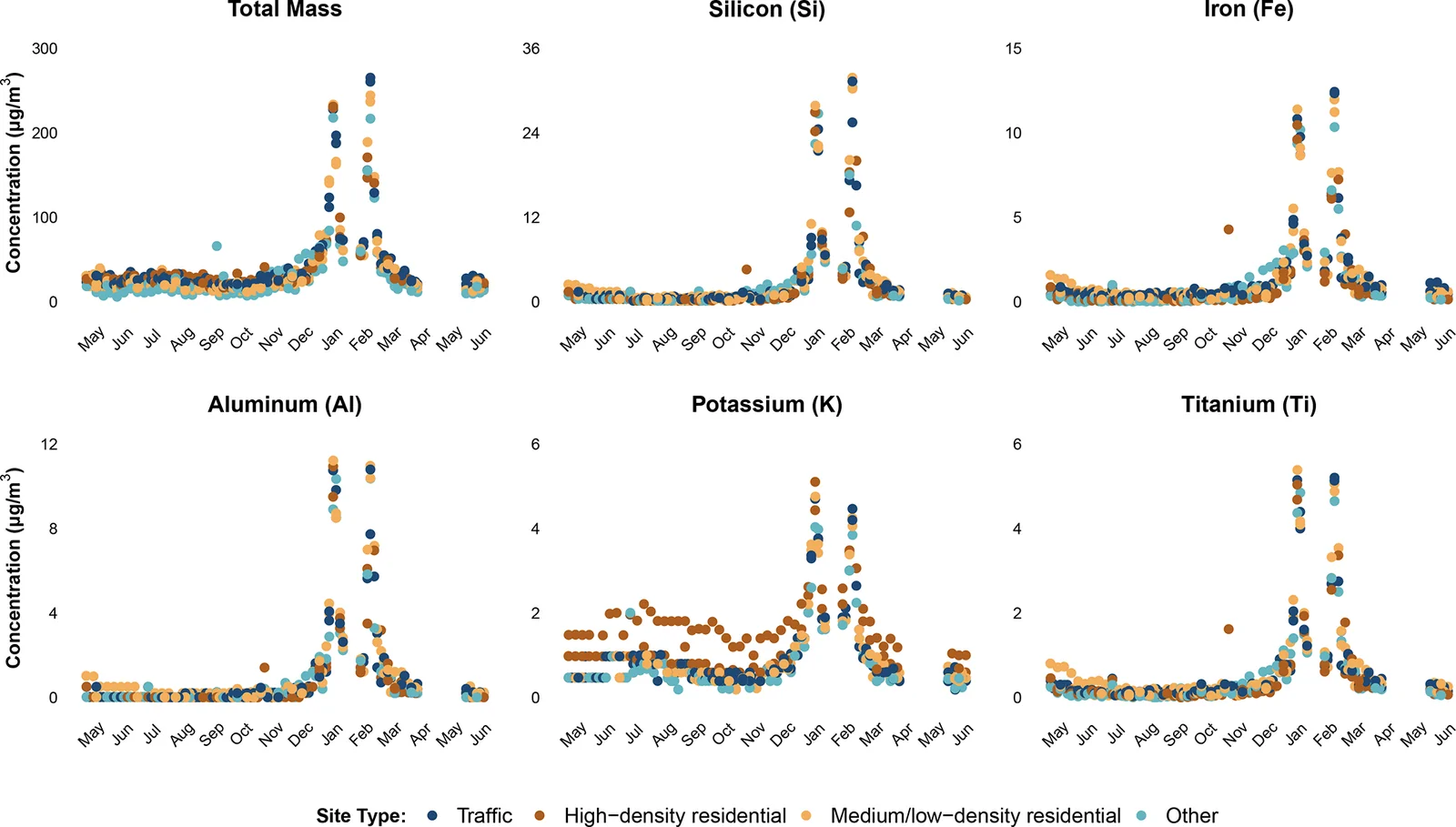
Seasonal variation in total PM_2.5_ mass and selected elements at the ten fixed sites. Traffic sites included N1 West (N1W), Tema Motorway (TMW), and Asylum Down (AD). High-density residential sites included Jamestown (JT) and Nima (NM), while medium/low-density residential sites included Ashaiman (ASH), East Legon (EL) and Taifa (TF). Other sites included coastal Labadi (LA) and the peri-urban background site, University of Ghana Hill (UGH).

During the non-Harmattan months, we observed strong correlations (≥0.8) among many of the crustal elements, including Al, Si, Ti, Fe, and Co (Figure 3). Notable correlations were also observed between the crustal elements Ca and Sc (0.96) and between Na and Cl (0.81), the latter of which is indicative of sea salt. Moderate associations were found between elements K and S (0.6) and between Pb, Zn, and Br (≥0.4), the latter of which are often found together in sources like non-exhaust vehicle emissions and solid/e-waste burning. Additionally, total PM_2.5_ mass was moderately correlated with Al, Si, K, Ca, Sc, Ti, Fe, and Co (≥0.4). During the Harmattan season, the correlations among crustal elements intensified (≥0.99), and the relationship between Zn, Pb, and Br also strengthened (≥0.5). Total PM_2.5_ mass also showed a stronger correlation with all elements, apart from Na, Cr, Cu, Zn, Br, La, and Pb during this period.

**Figure 3:**
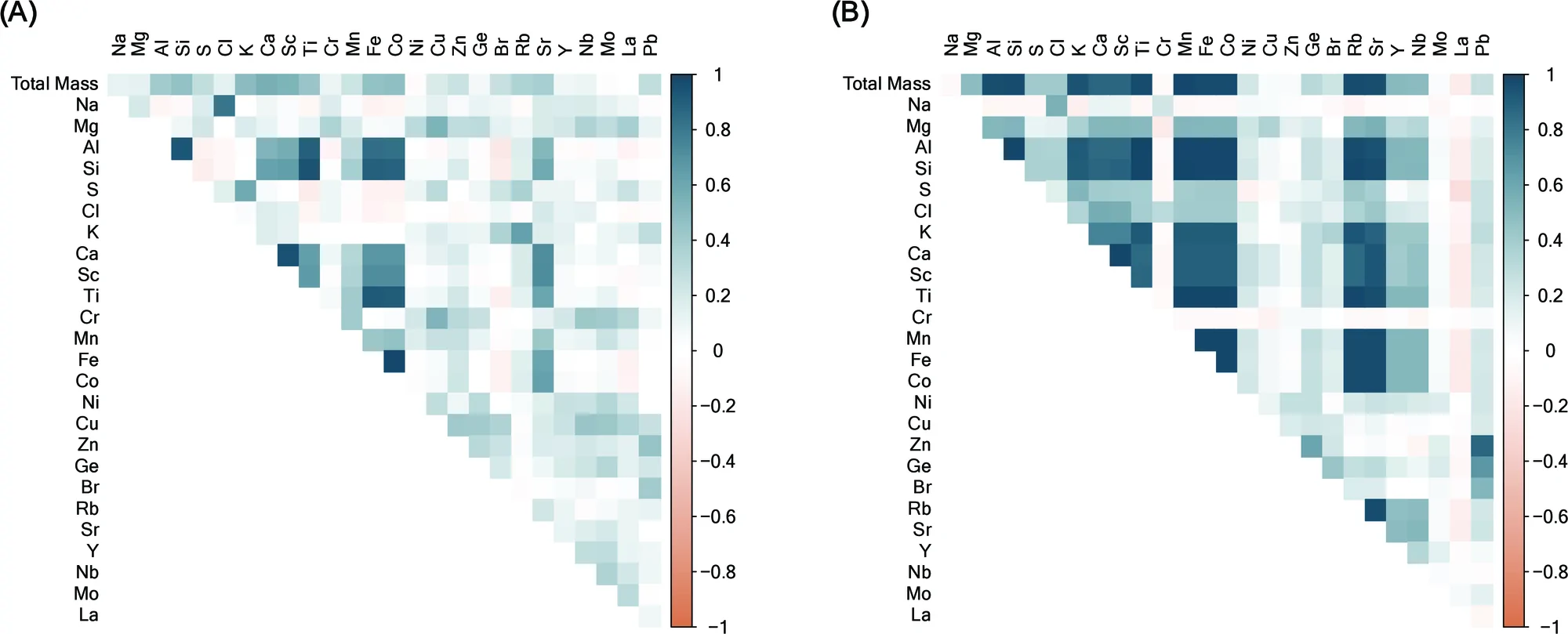
Pearson’s correlations between total PM_2.5_ mass and its elements during the (A) non-Harmattan and (B) Harmattan seasons.

### 3.2 Source Contributions to PM_2.5_ Pollution

#### 3.2.1 Performance of PMF Models

Bootstrap diagnostics indicated that the selected PMF solutions were generally stable, with most factors demonstrating high mapping percentages and minimal factor swapping. A small number of factors (n = 7 across all models) exhibited moderate stability (mapping ∼ 60-75%), likely reflecting overlap between compositionally related source profiles rather than substantive model instability. Additionally, most species exhibited strong S/N ratios (Table S3). Crustal elements (Al, Si, Ca, Ti, Fe) and several predominantly anthropogenic sources (K, S, Br), consistently demonstrated high S/N ratios across both seasons. In contrast, several trace elements (Cr, Ni, Cu, Ge, Y, Nb, Mo) exhibited low S/N ratios, in some cases approaching zero, suggesting limited temporal variability and reduced role in factor discrimination. These patterns were broadly consistent across fixed-site, rotating-site, and season-stratified models, supporting robustness of species selection despite differences in spatial and temporal model structure.

Q_true_/Q_exp_ ratios for the non-Harmattan fixed-site models ranged from 0.97 to 1.98, indicating generally good model fit (Table S3). For the full GAMA models, Q_true_/Q_exp_ values were 1.94 for the non-Harmattan period and 1.46 for the Harmattan period. Model performance was comparatively weaker for the rotating site classifications, with two site categories yielding Q_true_/Q_exp_ ratios >2, although these remained the best-fitting solutions among the factor configurations evaluated. For the fixed-site Harmattan models, Q_true_/Q_exp_ ratios ranged from 0.45 to 1.23, suggesting some degree of overfitting in selected models. However, in certain instances, these solutions were retained to preserve consistency in source profiles that would enable direct comparison across seasons.

#### 3.2.2 Fixed (Yearlong) Sites

In the non-Harmattan PMF models, we identified 4-6 potential sources of PM_2.5_ pollution across the ten yearlong monitoring sites: biomass burning, solid and electronic waste burning, traffic emissions and road dust, construction dust, crustal dust, and sea salt (Figure 4). Biomass burning was distinguished primarily by elevated concentrations of K.^19,20^ Solid and electronic waste combustion also showed elevated K, but at lower concentrations than biomass burning, and were further characterized by high concentrations of Br, Zn, and Pb.^21–23^ Traffic emissions and road dust were identified by the presence of Si, Al, Fe, and Ti that was often accompanied by Zn, Cu, and trace amounts of Pb.^24,25^ This profile differed from crustal dust, which was characterized by much higher concentrations of Al, Si, Fe, and Ti, typically alongside Ca and Mg.^26^ Construction dust was distinguishable from crustal sources due to the relatively higher abundance of Ca and/or Mg, which are both commonly associated with concrete and cement-based materials.^27,28^ Finally, sea salt was identified by high concentrations of Na and Cl, along with elevated S, which is indicative of aged sea salt aerosols.

**Figure 4:**
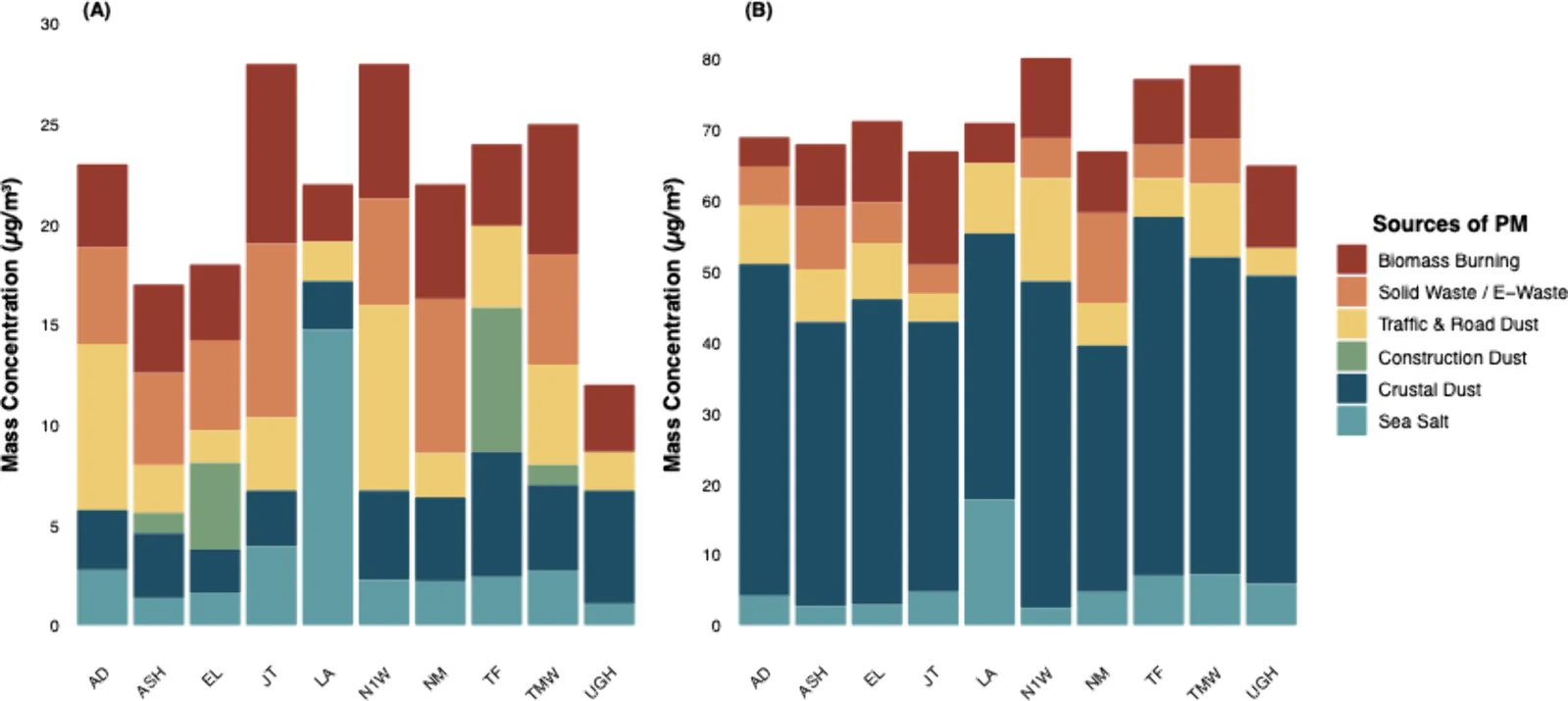
Contributions of sources to PM_2.5_ pollution across ten fixed monitoring sites during the (A) non-Harmattan and (B) Harmattan seasons. Source contribution percentages represent the proportion of the measured elemental PM_2.5_ mass apportioned to each PMF factor. These percentages are displayed in conjunction with total gravimetric PM_2.5_ concentrations to provide context regarding ambient PM_2.5_ levels across the study area. The ten fixed sites include Asylum Down (AD), Ashaiman (ASH), East Legon (EL), Jamestown (JT), Labadi (LA), N1 West (N1W), Nima (NM), Taifa (TF), Tema Motorway (TMW) and University of Ghana Hill (UGH).

On average, biomass burning contributed approximately 23% to total elemental PM_2.5_ mass across sites, though this contribution varied substantially from 13% in LA (ocean site in Labadi) to 32% in JT (a known high biomass-use indigenous Ga community). Solid and electronic waste combustion was another major contributor, making up an average of 18% of total elemental PM_2.5_ mass, with highest contributions in Nima (NM) (35%), Jamestown (JT) (31%), and Ashaiman (ASH) (27%), but notably absent at Labadi (LA), Taifa (TF), and the background site at the University of Ghana (UGH). At LA, TF, and UGH, other sources were more prominent; crustal dust accounted for a larger share of elemental PM_2.5_ mass at TF and UGH, contributing 25% and 47%, respectively. Sea salt dominated at the coastal site in LA, accounting for 67% of elemental PM_2.5_ mass.

Traffic emissions and road dust were especially influential at sites located near major roadways, contributing 36% at Asylum Down (AD), 33% at N1West motorway (N1W), and 20% at Tema Motorway (TMW). A distinct crustal source, likely associated with construction dust, also emerged at four sites. While this source represented a relatively small fraction of total elemental PM_2.5_ mass at ASH (6%) and TMW (3%), it appeared as a dominant contributor at East Legon (EL) (24%) and TF (30%), both of which had active construction projects during the monitoring period. Notably, this source did not emerge during the dusty Harmattan season, likely due to the overwhelming influence of regional crustal dust, which made it difficult to distinguish construction-related contributions. Though biomass burning, solid and electronic waste combustion, traffic emissions and road dust, and sea salt continued to appear as sources during Harmattan, their contributions to elemental PM_2.5_ substantially reduced. Instead, crustal dust dominated across all sites during this season, accounting for 52–68% of total elemental PM_2.5_ mass (Figure 4).

#### 3.2.3 Rotating (Weeklong) Sites

Our site-type PMF models, which incorporated data from 121 rotating (weeklong) monitoring locations, identified 5-6 potential PM_2.5_ sources (Figure S1). These sources largely mirrored those found in the fixed-site models, except for construction dust, which did not emerge as a distinct source. Instead, metal processing appeared as a potential source, but only within CBI sites, contributing 9.7% to total elemental PM_2.5_ mass. This source was characterized by elevated levels of Zn, Pb, Ni, Al, along with trace amounts of Mo, La, and Nb, yet lacked the elevated concentrations of K and Br that typically signify combustion. CBI sites also had substantial contributions from biomass burning (22%) as well as traffic emissions and road dust (20%).

Sites in both high-density and medium/low-density residential communities exhibited similar contributions from solid and electronic waste combustion (24-28%). However, the contribution of biomass burning was unexpectedly higher in medium/low-density residential areas than high density sites (23-30% vs 12%). In contrast, high-density sites had a noticeably higher contribution from traffic emissions and road dust (20%), potentially reflecting more extensive and congested road networks in these environments (Figure S1). The contribution of crustal sources was relatively consistent across site types, except for peri-urban/background areas, where it accounted for a much larger portion of total elemental PM mass (43%).

#### 3.2.4 Combined GAMA Models

To provide a broad overview of PM_2.5_ sources across the entire GAMA, we analyzed all samples collected during the study period together, regardless of the site type but separated by season. In the non-Harmattan period, biomass burning contributed the largest to elemental PM_2.5_ mass (27%), followed by traffic and road dust (26%), crustal dust (16%), sea salt (15%), construction dust (9%) and solid/e-waste burning (7%). Source contributions were generally similar during the Harmattan period, however, crustal dust dominated, contributing 61% of total elemental PM_2.5_ mass, and construction dust did not emerge as a distinct source (Figure 5).

**Figure 5:**
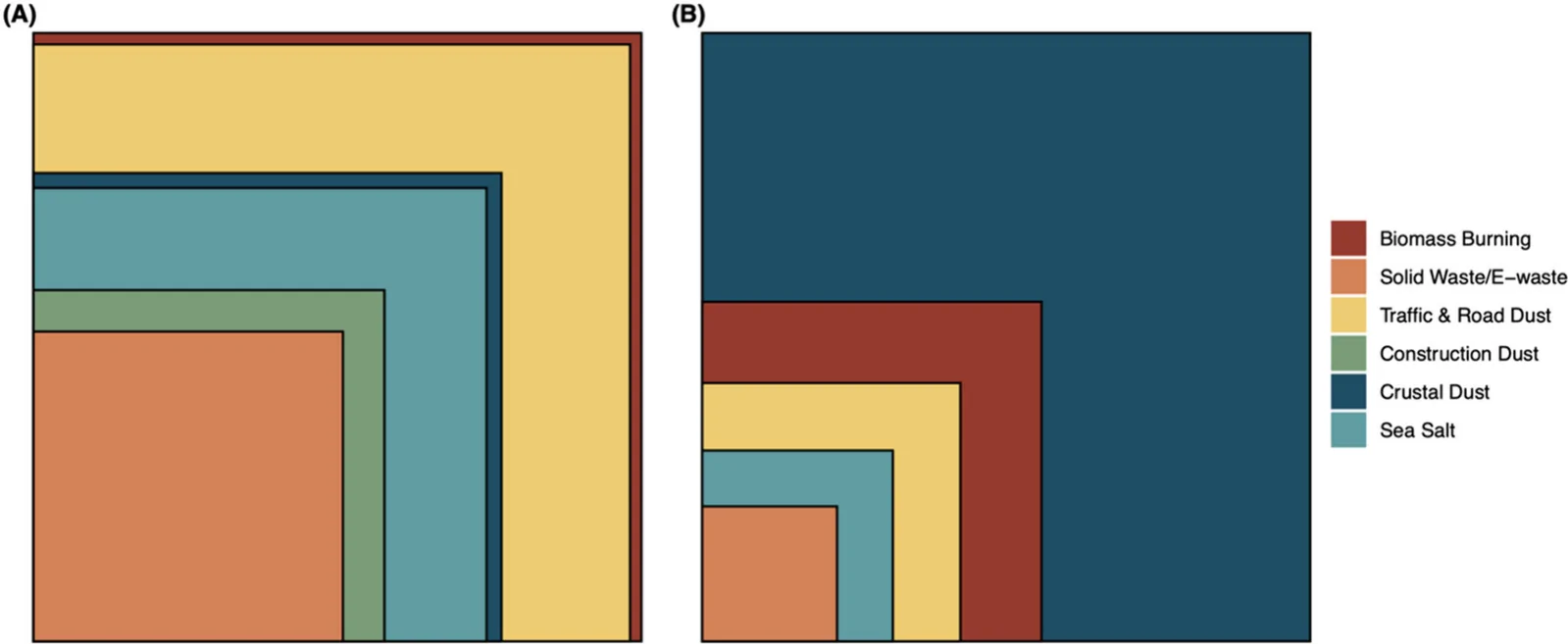
Contributions of sources to PM_2.5_ mass concentrations across all study sites (including repeated measurements at fixed sites; n = 603) during the (a) non-Harmattan and (b) Harmattan seasons. Source contribution percentages represent the proportion of the measured elemental PM_2.5_ mass apportioned to each PMF factor. These percentages are displayed in conjunction with total gravimetric PM_2.5_ concentrations to provide context regarding ambient PM_2.5_ levels across the study area.

### 3.3 Decade Long Change in PM_2.5_ Composition and Sources

The previous studied conducted by Zhou and colleagues identified 5-6 potential sources of PM_2.5_ across the four legacy sites (AD, EL, JT, and NM), including fresh biomass burning, aged biomass burning, solid waste burning, traffic and road dust, sea salt, and crustal dust. To facilitate direct comparison between studies, we combined the previously distinguished fresh and aged biomass factors into a single biomass burning source. Similarly, at site EL, we combined the construction dust and crustal dust factors identified in our current study. PM_2.5_ mass concentrations at three of the four sites (AD, NM, and JT) decreased by 35-60% over the decade, consistent with overall reductions in PM pollution across the city during this period. In contrast, EL exhibited a 21% increase, likely reflecting more recent and rapid urbanization and commercialization at this site relative to the others.^14^ Notably, the relative contributions of individual sources at these sites have also shifted over the decade (Figure 6). In the earlier study, biomass burning accounted for 35-45% of total elemental PM_2.5_ mass across the four neighborhoods, followed by traffic and road dust (10-30%) and solid waste burning (0-11%). In the current study, biomass burning remains an important source but has declined significantly to 18-32% (mean difference = 15.5%; 95% CI: 5.2-25.8%; p = 0.017). Over the same period, contributions from solid and electronic waste burning increased significantly to 21-35% (mean difference = 23.0%; 95% CI: 15.9-30.0%; p = 0.002). Additionally, the proportion of PM_2.5_ attributed to the combined traffic and road dust factor, and to crustal dust, remained relatively stable across the decade.

**Figure 6:**
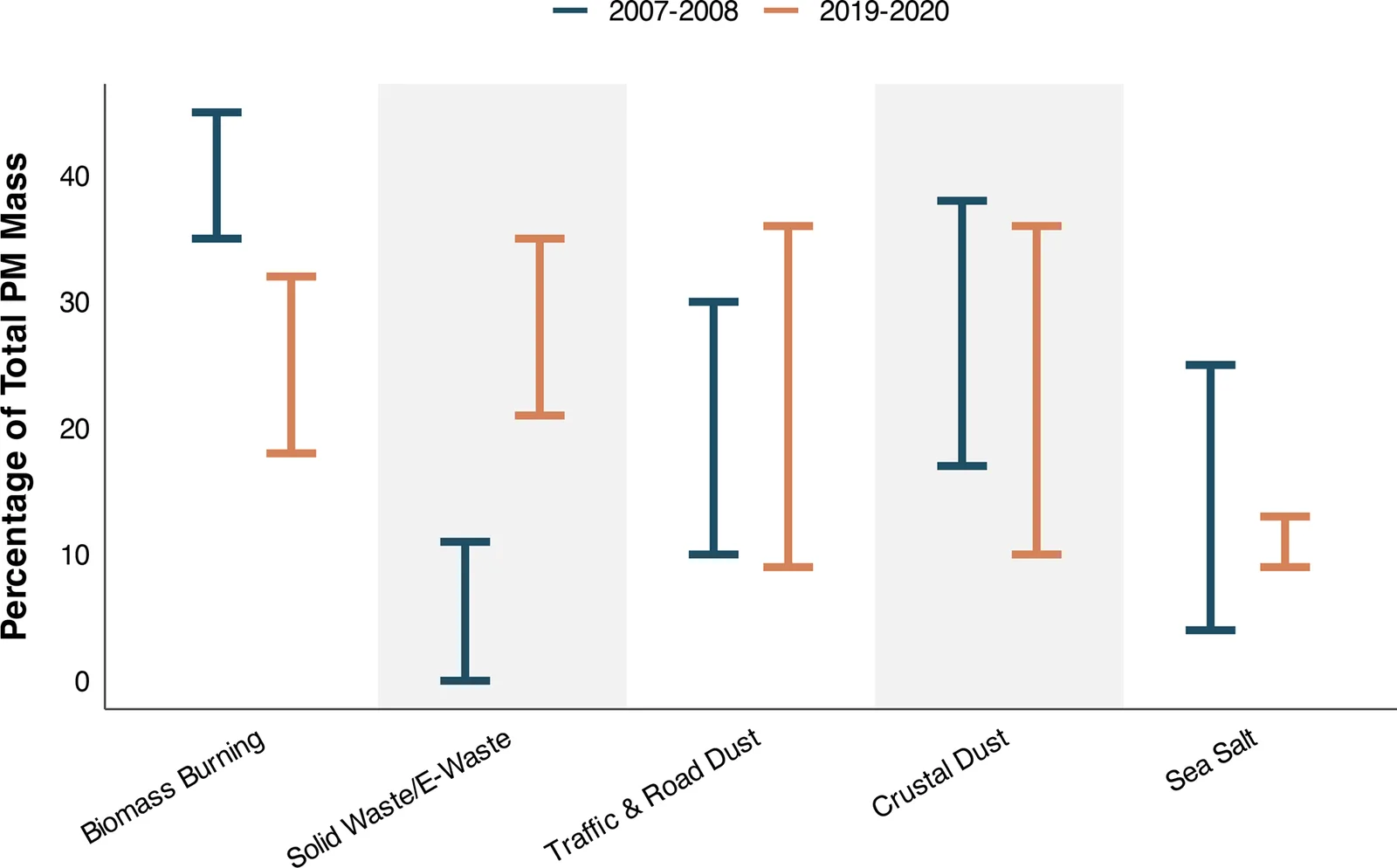
Comparison of source contributions between 2007-2008 and current 2019-2020 studies across the four monitoring sites: AD, EL, JT and Nima. Bars represent the range of percent contributions.

## 4 Discussion

There is growing evidence that the ongoing urbanization and economic expansion across SSA are shifting the dominant sources of urban air pollution, with potentially substantial implications for PM_2.5_ levels, composition, and the relative contributions of major emission sources. However, data on the elemental composition and source contributions of PM_2.5_ in the region remain scarce, limiting efforts to design targeted, sector-specific emission reduction strategies. In particular, no study in SSA has, to our knowledge, empirically examined changes in urban PM_2.5_ elemental composition and source contributions over time. Our work represents one of the largest and most spatially resolved efforts to characterize elemental PM_2.5_ composition and sources in an SSA city and uniquely examines changes in source contributions over approximately a decade in Accra, Ghana. Overall, concentrations of elements indicative of biomass combustion and vehicular activity were highest in densely populated residential neighborhoods and heavy-traffic areas, respectively. Pronounced seasonal differences were evident for several elements related to crustal dust during the Harmattan season, where they accounted for more than half of the total elemental PM_2.5_ mass. During the non-Harmattan season, when local emissions dominate, elemental PM_2.5_ mass was apportioned primarily to (in decreasing order of contributions) biomass burning, traffic emissions and road dust, solid and electronic waste combustion, construction dust, crustal dust, and sea salt. Comparison with a 2007-2008 study at four of the same monitoring sites indicates that contributions from traffic and road dust and crustal dust have remained relatively stable over the past decade, while biomass burning has declined significantly. At the same time, solid and electronic waste burning has risen substantially and now approaches the contributions of biomass burning and traffic.

Our findings align with the previous source apportionment study conducted in Accra, which also identified biomass burning, traffic and road dust, solid waste burning, crustal dust, and sea salt, as key contributors to ambient PM_2.5_.^12^ However, the present study is larger in scope and provides updated and more detailed insights into how the relative contributions of these sources have evolved over time. Compared to the earlier study, which reported biomass burning as the dominant contributor and solid waste burning as a minor one, we observed a notable decline in biomass burning contributions and a corresponding increase in those from solid waste burning. Despite this shift, the overall consistency of source profiles underscores the persistence of these major contributors in the region. Our results are similar to the few other source apportionment studies conducted in West Africa, which have identified related dominant sources: traffic (motor vehicles and resuspended road dust), solid fuel combustion, and crustal dust in Bamako, Mali, and traffic, crustal dust, sea salt, and industrial emissions in Dakar, Senegal.^29,30^

Across the city of Accra, PM_2.5_ mass concentrations have substantially declined over the past decade and now appear to have plateaued at levels above those seen in major cities in high-income countries (e.g. New York, USA and London, UK) but far below those in Asian cities (e.g. Beijing, China and Delhi, India).^12^ Our findings suggest that this decline is largely driven by a reduction in the proportion of homes using biomass for cooking, particularly the use of fuelwood in low-income, densely populated neighborhoods, where the largest decreases in PM_2.5_ were observed. This trend aligns with national shifts away from traditional biomass (fuelwood and charcoal) fuels, supported by government initiatives promoting liquefied petroleum gas (LPG), including the distribution of free equipment and subsidies.^31,32^ Despite this progress, however, biomass use is still prevalent and remains a key contributor to particle pollution in the city. Recent census data indicate that roughly 44% of urban households still rely on charcoal (processed wood) and nearly 13% on wood fuel as their primary cooking fuels.^33^ Thus, while meaningful progress has been made, further targeted interventions are necessary to accelerate the transition to cleaner household energy sources and promote additional reductions of air pollution in Accra.

In contrast to the decline in biomass emissions, contributions from solid waste and e-waste burning have increased, with source contributions at the legacy monitoring sites now approaching those of biomass burning and traffic. We emphasize, however, that this trend is not representative of the entire city but rather is specific to the four legacy monitoring locations. Our pooled, citywide non-Harmattan model, which incorporated all fixed and rotating sites, estimated that solid/e-waste burning contributed a substantially smaller share of PM_2.5_ (∼7%) than either biomass burning (∼27%) or traffic (∼26%). This discrepancy likely reflects the spatial concentration of informal waste and e-waste burning activities within specific neighborhoods rather than a citywide phenomenon. We therefore interpret the legacy-site trend as strong evidence that waste burning has become a locally dominant PM_2.5_ source in certain high-density neighborhoods over the past decade, whereas the pooled model indicates that, when averaged across the metropolis, waste burning remains a smaller contributor than biomass burning or traffic. Nevertheless, the marked increase in solid and e-waste burning at the legacy monitoring locations underscores the need for greater attention to emissions from these sources, particularly in low-income and densely populated communities.

On July 1, 2021, approximately one year after the conclusion of our monitoring campaign, the Ghanaian government demolished the Agbogbloshie scrapyard, the major site for informal e-waste processing. However, this action has led to the emergence of several new informal e-waste burning sites across Accra.^34^ Future studies should monitor and assess how these evolving waste-burning activities continue to influence the composition of PM_2.5_ across the GAMA. In parallel, policy efforts must focus on this growing issue by establishing a comprehensive national strategy that raises public awareness of the health risks associated with e-waste burning, restricts the importation of electronic waste, and enforces regulations targeting informal burning operations along with comprehensive solid waste management plans in the region.^6^

It is well established that the vehicle fleet in Accra has grown significantly over time, leading to increased traffic congestion and related emissions.^35^ Nitrogen dioxide (NO_2_), a pollutant closely linked to road traffic and commonly used as a marker of vehicle emissions in urban environments, has risen by approximately 50-150% over the past decade.^36,37^ However, our results indicate that the contribution of traffic-related sources to particle pollution in the city has remained relatively stable over the same period at four legacy sites. This finding should not necessarily be interpreted as evidence that vehicle emissions themselves have remained unchanged, as the source factor identified represents a combination of both exhaust-related and non-exhaust traffic emissions. While vehicle activity and exhaust emissions may have increased, these changes may have been offset by reductions in resuspended road dust associated with increased road paving. Additionally, elemental analysis primarily captures non-exhaust traffic markers and provides limited information on carbonaceous compounds of vehicle exhaust, such as volatile organic compounds (VOCs) and polycyclic aromatic hydrocarbons (PAHs), which have been shown to be more reliable indicators of combustion-related traffic emissions.^38,39^ This suggests that source apportionment based solely on elemental composition may not fully resolve changes in traffic exhaust emissions, particularly when exhaust and road dust contributions are combined into a single factor. That said, it is also possible that the vehicle fleet in Accra has become cleaner as it has expanded, particularly following the publishing of standards for Environment and Health Protection – Requirements for Motor Vehicle Emissions (GS 1219) in 2018.^40^ Taken together, while our results suggest that the elemental contribution of the combined traffic and road dust source has remained stable, traffic-related emissions may have changed over time. Future studies incorporating gaseous pollutants and particulate organic compounds are needed to better characterize temporal trends in traffic emissions.

Our analysis also revealed that crustal dust contributed substantially to PM_2.5_ levels, accounting for more than 50% of total elemental PM_2.5_ mass during the Harmattan season. While this meteorological event may initially seem beyond the scope of intervention, several policy strategies have been proposed to reduce its impact. One key approach involves scaling up mitigation efforts such as ecosystem restoration, afforestation, and soil or sand stabilization.^41^ In addition, sustainable agricultural practices, such as maintaining natural vegetation on croplands during the dry season, have been shown to reduce the impact of wind erosion and help trap Harmattan dust.^42^ While these measures can reduce local dust emissions, Harmattan dust is transported across the entire region, affecting multiple countries, thus highlighting the need for coordinated, regional-scale cooperation to address transboundary dust pollution. Beyond land management, strengthening dust monitoring systems can help ensure timely public notifications during high-dust events, allowing urban residents, especially sensitive subpopulations like children, pregnant women, elderly, and those with preexisting conditions, to take protective measures such as staying indoors and wearing masks. These policy measures have become increasingly urgent, especially as climate change is projected to make the Harmattan season longer, hotter, and drier in the coming decades.^43^

More broadly, these findings highlight the importance of moving beyond measurements of total PM_2.5_ mass to better understand the sources and composition driving population exposure. Because the toxicity and health implications of PM_2.5_ may vary by composition and emission source,^44,45^ source-specific characterization is essential for identifying interventions that maximize public health benefits. The increasing contribution of solid and e-waste burning is of particular concern, as these emissions are enriched in Pb and other toxic trace metals associated with adverse neurodevelopmental and cardiovascular outcomes. Notably, the greatest increases in these sources were found to be in low-income, densely populated neighborhoods that have previously been identified as environmental justice hotspots in Accra.^46,47^ Similarly, the traffic-related factor, though representing a composite of traffic emissions and resuspended road dust, reflects exposures that disproportionately impact those living along major transportation corridors^37,48^ and have been linked to respiratory and cardiovascular morbidity.^49^ During the Harmattan season, the predominance of crustal dust further highlights the need for seasonal public health messaging and exposure mitigation strategies, particularly for vulnerable populations who may be at increased risk of respiratory illness. In rapidly growing urban areas, where resources for air quality management are limited, prioritizing interventions targeting dominant and potentially modifiable sources may provide a more effective strategy for reducing exposure and protecting population health.

### 4.1 Strengths and Limitations

Our study has several notable innovations and strengths. To our knowledge, this is one of the largest and longest studies to characterize PM_2.5_ composition and quantify contributions from major sources in a SSA city, a region experiencing high levels of air pollution but with limited measurement data to inform policy. Furthermore, the study design, which included repeated measurements at fixed sites and one-time measurements across diverse geographical areas within the GAMA, enabled us to assess both temporal trends while incorporating broader geographic information into several of our PMF models. This approach provided insight into how source contributions may differ by land use type and across the GAMA more broadly. In addition, this spatially resolved dataset is particularly valuable, as it provides a foundation for future modeling efforts that could predict elemental concentrations and source contributions across the entire GAMA, including areas not directly measured. Such models would enable more comprehensive exposure assessment for future epidemiological studies investigating the health impacts of specific PM_2.5_ components and sources. Beyond its methodological contributions, our study has strong policy relevance. Most prior studies in SSA have focused solely on PM_2.5_ mass rather than identifying specific sources, which limits the development of targeted mitigation strategies. While total PM_2.5_ mass data are useful for quantifying pollution levels, such information alone provides little guidance for designing effective interventions, particularly in low-resources settings where broad efforts to reduce overall PM_2.5_ may be financially or logistically unfeasible. Thus, by identifying the major contributing sources, our study provides the evidence needed to prioritize targeted and cost-effective emission control strategies. These insights are not only critical for guiding policy in Accra but also provide a framework for understanding and addressing urban air pollution challenges in other rapidly growing cities across West Africa and low-and middle-income countries, where similar challenges from urbanization, informal waste management, household combustion, and transportation growth are increasingly shaping air quality.

While this study has many strengths, several limitations should be acknowledged. As with any receptor modeling approach, PMF results are subject to uncertainty arising from analytical error and assumptions in uncertainty estimation, particularly for elements with concentrations near detection limits. In addition, some PMF factor profiles reflected mixed or overlapping source signatures, making it challenging to attribute them unambiguously to specific emissions sectors (e.g., distinguishing between solid waste burning and e-waste burning, or between vehicle exhaust and resuspended road dust). For this reason, these sources were combined into broader categories; for solid and e-waste specifically, it is likely that most of this contribution reflects solid waste burning. Additionally, differences in XRF instrumentation and calibration procedures between the current study and prior work may introduce uncertainty when interpreting changes in estimated source contributions over time. However, the primary elemental tracers used to identify major source factors, including elements for solid/electronic waste burning, were also measured in the previous study, and source interpretations were based on consistent elemental profiles rather than newly detected species. Furthermore, this study assessed only the elemental composition of PM_2.5_ and did not include organic compounds, elemental carbon, or secondary inorganic species such as nitrate and ammonium. While elemental tracers provide valuable information for source identification, the absence of these additional chemical components limits the ability to fully quantify contributions to total PM_2.5_ mass. Sources with large carbonaceous or secondary aerosol fractions, such as biomass burning and vehicle emissions, may be less completely represented than sources dominated by inorganic mineral components, such as crustal dust. Organic species and other non-elemental components can also provide stronger source specificity, particularly for combustion-related sources, and offer additional insight into the toxicological relevance of different pollution sources. Thus, the source contributions represented here should be interpreted as the distribution of measured elemental PM_2.5_ mass rather than a complete mass closure of total gravimetric PM_2.5_. Finally, while the findings provide important insights into urban air pollution sources in Accra and may be relevant to other rapidly growing urban areas in West Africa and other low- and middle-income regions, source contributions will likely vary across locations with different industrial activities, transportation systems, energy use, and waste management practices. Therefore, local measurements and source characterization remain essential for developing effective, context-specific emission reduction strategies.

## 5 Conclusion

Particle pollution in Accra has decreased significantly over the past decade, and our findings suggest that this reduction is largely driven by decreases in biomass burning. While promoting a transition from biomass use to cleaner alternatives like LPG remains a key policy strategy for reducing PM_2.5_ pollution, increasing attention must also be directed toward emissions from solid and electronic waste burning. In many high-density neighborhoods, contributions from these sources have risen to levels approaching those of biomass burning and traffic, highlighting the need for improved waste management/collection services to prevent open trash burning, as well as restrictions on e-waste importation and stronger regulatory efforts to curtail e-waste burning. Altogether, these findings underscore the importance of targeted, multi-source interventions to reduce PM_2.5_ and protect public health in rapidly urbanizing SSA.

## Supplementary Material



## Data Availability

Environmental measurement data is available through the open-source platform, Zenodo. Those interested in accessing the data should direct any questions to Raphael E. Arku [rarku@umass.edu].

## Notes

Summary of method detection limits (MDL); tables of Q statistics (Q_robust_, Q_true_, and Q_true_/*Q*_exp_) and signal-to-noise (S/N) ratios; figure of PM_2.5_ source contributions at rotating sites (PDF)
