# Supplementary material for: Shifting Dominant Air Pollution Sources in Sub-Saharan Africa’s Changing Urban Environment: Analysis of Elemental Composition and Source Contributions of Ambient Fine Particulate Matter (PM2.5) Pollution in Accra, Ghana
Source: Environ Health Perspect. Author manuscript; Available in PMC 2026 Oct 29. (PMC7619642; doi:10.1021/EHP.6c00323)
Supplement: Supplementary file 1 [file EMS221681-supplement-Supporting_Information.pdf]

Shifting dominant air pollution sources in sub-Saharan Africa's changing urban environment: Analysis of elemental composition and source contributions of ambient fine particulate matter ($PM_{2.5}$) pollution in Accra, Ghana

Carissa L Lange[1], Aaron J Specht[2], Abosede S Alli[1], Sierra N Clark[3], James Nimo[4,5], Thomas Grier[2], Raji Balasubramanian[6], Michael Brauer[7,8], Allison F Hughes[4], Majid Ezzati[9-12], Raphael E Arku[1,*]

[1]Department of Environmental Health Sciences, University of Massachusetts, Amherst, MA, USA
[2]School of Health Sciences, Purdue University, Lafayette, IN, USA
[3]Department of Population Health & Policy, School of Health and Medical Sciences, City St George's, University of London, London, UK
[4]Department of Physics, University of Ghana, Legon, Accra, Ghana
[5]Department of Environmental & Sustainable Engineering, State University of New York, Albany, NY, USA
[6]Department of Biostatistics & Epidemiology, University of Massachusetts Amherst, Amherst, MA, USA
[7]School of Population and Public Health, The University of British Columbia, Vancouver, CA
[8]Institute for Health Metrics and Evaluation, University of Washington, Seattle, USA
[9]Department of Epidemiology and Biostatistics, School of Public Health, Imperial College London, London, UK
[10]Regional Institute for Population Studies, University of Ghana, Accra, Ghana
[11]MRC Centre for Environment and Health, School of Public Health, Imperial College London, London, UK
[12]Imperial Global Ghana, Accra, Ghana

The supplementary materials contain a combination of experimental details, materials, methods, and results, including a summary of the method detection limits (MDL) used for ED-XRF analysis, signal-to-noise (S/N) ratios for $PM_{2.5}$ species, Q statistics (Q_{robust}, Q_{true}, and Q_{true}/Q_{exp}) across PMF models, and source contributions to $PM_{2.5}$ elemental mass across rotating site types.

Downloaded from pubs.acs.org/ev/hpaz/article-supplement/5448177/pdf/hp6c00323_si_001/ by guest on 24 October 2026

Table S1. Summary of Method Detection Limits (MDL).

Element	Detection limit (ng/cm^2)
Na	160.59
Mg	156.92
Al	7.99
Si	1.68
S	3.11
Cl	3.16
K	0.30
Ca	1.38
Sc	0.51
Ti	1.42
Cr	12.84
Mn	2.83
Fe	22.56
Co	3.63
Ni	0.54
Cu	12.10
Zn	5.84
Ge	0.45
Br	0.23
Rb	0.23
Sr	1.74
Y	3.04
Nb	4.3
Mo	3.90
La	1.11
Pb	4.45

Downloaded from pubs.acs.org/evhpaz/article-supplement/5448177/pdf/hp6c00323_si_001/ by guest on 24 October 2026

Table S2. Q Statistics (Q_{robust}, Q_{true}, and Q_{true}/Q_{exp}) for all models. Q_{true} reflects model fit including all data points, whereas Q_{robust} downweighs extreme residuals to reduce the influence of outliers. Values of Q_{true}/Q_{exp} near 1 indicate good model fit, whereas values substantially greater than 1 suggest underfitting and values substantially less than 1 suggest potential overfitting.

	**Non-Harmattan**			**Harmattan**		
**Site**	**Q(Robust)**	**Q(True)**	**Q(true)/Qexp**	**Q(Robust)**	**Q(True)**	**Q(true)/Qexp**
	Full GAMA Models					
All sites	15220.47	16474.44	1.946413016	4590.8795	5090.9485	1.46207605
	Fixed Site Models					
AD	777.4687	787.88825	1.399446279	103.468745	103.46868	0.7185325
ASH	790.018	791.3045	1.510123116	201.0147	201.0146	1.22569875
EL	729.65245	747.86535	1.483859819	178.967	189.01665	1.1525406
JT	860.8747	868.4683	1.487103242	104.79495	104.7924	0.727725
LA	924.09715	1034.7815	1.837977827	95.48289	95.482715	0.58221167
N1W	561.9818	615.71675	1.13183226	73.76132	73.761295	0.51223121
NM	886.4775	902.95195	1.546150613	160.11025	160.10775	0.86544729
TF	563.899	564.70275	0.966956761	64.88279	64.882495	0.45057287
TMW	765.28235	823.36205	1.513533169	36.460235	36.46016	0.56092554
UGH	1050.9595	1112.5755	1.97615543	185.24345	191.8857	1.19928563
	Rotating Site Models					
Background	217.3904	217.3904	0.957666957	-	-	-
CBI	426.9323	482.1085	2.363276947	-	-	-
High-dens	351.15645	351.1576	1.083819759	-	-	-
Med/low-dens1	590.4913	670.5809	2.205858219	-	-	-
Med/low-dens2	157.39095	157.39085	1.558325255	-	-	-

Downloaded from pubs.acs.org/ev/hpaz/article-supplement/5448177/pdf/hp6c00323_si_001/ by guest on 24 October 2026

Table S3. Signal-to-noise (S/N) ratios for $PM_{2.5}$ species across PMF models. For the fixed-site models, S/N ratios were averaged across the 10 monitoring sites separately for the Harmattan and non-Harmattan periods. For rotating-site models, which only included non-Harmattan period data, S/N ratios were averaged across the five site groupings. For full GAMA models, S/N ratios are reported directly for the Harmattan and non-Harmattan models.

	**Signal-to-noise (S/N) ratios**				
Species	Fixed Sites (non-Harmattan)	Fixed Sites (Harmattan)	Rotating Sites (non-Harmattan)	All Sites (non-Harmattan)	All Sites (Harmattan)
Na	0.3	0.1	0.1	0.3	0.1
Mg	2.0	1.4	1.4	1.9	1.4
Al	2.7	7.8	3.6	2.9	7.9
Si	9.0	9.0	9.0	9.0	9
S	9.0	9.0	9.0	9.0	9
Cl	7.3	7.5	7.5	7.4	7.3
K	9.0	9.0	9.0	9.0	9
Ca	8.5	9.0	8.7	8.5	9
Sc	7.2	8.6	7.4	7.3	8.6
Ti	8.6	9.0	8.6	8.6	9
Cr	0.0	0.0	0.0	0.0	0
Mn	0.0	1.5	0.0	0.0	1.4
Fe	5.5	8.4	5.9	5.6	8.4
Co	1.0	5.4	1.1	1.0	5.3
Ni	0.0	0.0	0.0	0.0	0
Cu	0.3	0.0	0.0	0.2	0
Zn	2.0	4.9	1.9	2.0	4.9
Ge	0.1	0.3	0.1	0.1	0.3
Br	8.5	8.9	8.4	8.4	8.8
Rb	1.6	5.4	2.0	1.7	5.2
Sr	0.1	3.1	0.1	0.1	3
Y	0.0	0.0	0.0	0.0	0
Nb	0.0	0.0	0.0	0.0	0
Mo	0.0	0.0	0.0	0.0	0
La	0.8	0.2	0.9	0.8	0.2
Pb	0.4	1.4	0.6	0.5	1.4

Downloaded from pubs.acs.org/ev/hpaz/article-supplement/5448177/pdf/hp6c00323_si_001/ by guest on 24 October 2026

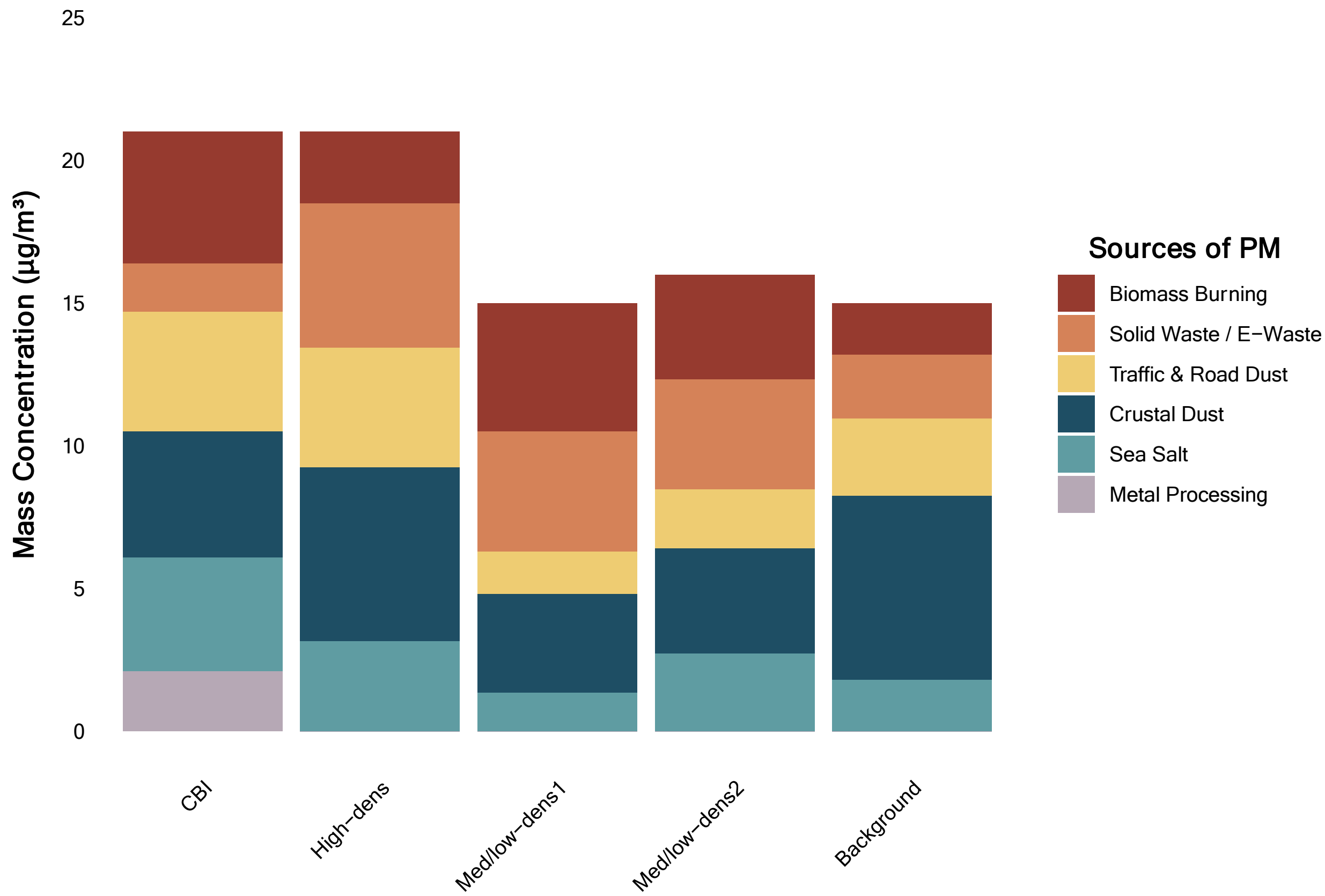

Figure S1. Contributions of sources to $PM_{2.5}$ mass concentrations across rotating monitoring site types during the non-Harmattan seasons.

Downloaded from pubs.acs.org/evhpaz/article-supplement/5448177/pdf/hp6c00323_si_001/ by guest on 24 October 2026
